# MiRNA-155 Regulates the Th17/Treg Ratio by Targeting SOCS1 in Severe Acute Pancreatitis

**DOI:** 10.3389/fphys.2018.00686

**Published:** 2018-06-08

**Authors:** Dongyan Wang, Maochun Tang, Pengfei Zong, Hua Liu, Ting Zhang, Yu Liu, Yan Zhao

**Affiliations:** ^1^Department of Gastroenterology, Tenth People’s Hospital of Tongji University, Shanghai, China; ^2^The Community Health Service Center of Nanxiang Town, Shanghai, China

**Keywords:** acute pancreatitis, miRNA-155, SOCS1, Th17, CD4^+^ T cells

## Abstract

Acute pancreatitis (AP) is a serious condition associated with intestinal barrier disruption or inflammation of the pancreatic tissue. Specific microRNAs are involved in the pathogenesis of AP, during which IL-17-producing CD4^+^ T helper (Th17) cells accumulate in the pancreas. In this study, significantly increased levels of miR-155 were detected in clinical samples from patients with AP, and overexpression of miR-155 correlated with severe AP (SAP). To identify the effect of miR-155 on T cell differentiation, we isolated CD4^+^ T lymphocytes and *in vitro* experiments showed that inhibition of miR-155 significantly reversed the stress-induced increase in the Th17/Treg ratio. The results also showed that miR-155 increased the Th17-mediated inflammatory response by targeting SOCS1. The interaction between miR-155 and the 3^′^-UTR of SOCS1 was confirmed by a dual luciferase reporter assay and RT-PCR. Experimental AP of varying severity was induced in BALB/c mice by caerulein hyperstimulation and miR-155 expression was found to increase with disease progression. Inhibition of miR-155 expression significantly improved the pathology of the pancreas. We also observed downregulation of expression of inflammatory factors, IL-17, SOCS1 and phosphorylated STAT1 after miR-155 inhibition. In summary, miR-155 regulates the Th17/Treg ratio by targeting SOCS1, most probably via direct binding to its 3^′^-UTR region, indicating that this microRNA may be a potential biomarker and/or therapeutic target for AP.

## Introduction

Acute pancreatitis (AP) is a condition characterized by sudden inflammation of the pancreas resulting from abnormal activation of digestive enzyme precursors (zymogens) within the pancreas, the most notable of which is trypsinogen. This dysregulation of digestive enzymes leads to autodigestion of the pancreas, inflammation, edema, vascular injury, and in some cases apoptotic or necrotic cell death. The severity of the disease course is determined by whether the predominant response to cellular injury is inflammation and apoptotic cell death (leading to mild, self-limiting, AP, in approximately 80% of cases) or necrotic cell death (leading to severe AP, or SAP, in approximately 20% of cases) (Bhatia, 2004). The inflammatory response involves the synthesis and secretion of inflammatory mediators, such as TNF-α, IL-1, and IL-6, by pancreatic cells and the recruitment of neutrophils to the pancreas (Norman, 1998). In SAP, necrosis of the pancreatic tissue occurs and nearby organs may also be affected. Patients suffer endotoxemia as a result of apical junction complex damage and intestinal barrier dysfunction (Ammori et al., 1999, Ammori, 2003; Rahman et al., 2003; Capurso et al., 2012) and in severe cases multiple organ failure and death, with a reported mortality rate of 30% (Juvonen et al., 2000; Clark and Coopersmith, 2007; Wu and Banks, 2013).

The cellular injury resulting from autodigestion of the pancreas during AP induces the accumulation of IL-17-producing CD4^+^ T helper (Th17) cells stimulating an inflammatory response that is the hallmark of this disease. In fact, a Th17/Treg imbalance is associated with various autoimmune and inflammatory diseases (Tzartos et al., 2008; Jamshidian et al., 2013, 2015; Lochner et al., 2015; Venkatesha et al., 2015). An increase in the Th17/regulatory T (Treg) cell ratio leads to considerable elevation in cytokine levels, which in turn, transduces signals via the Janus kinase (JAK)/signal transducer and activator of transcription (STAT) pathway. The SOCS (suppressor of cytokine signaling) protein family have been reported to target the JAK/STAT pathway modulating the inflammatory response (Fujimoto and Naka, 2010; Wu et al., 2014). However, the interrelationships between these signaling molecules is complex with different SOCS proteins targeting different STAT regulators, which in turn affect different cytokines, thereby leading to different outcomes. It is well established in the literature that SOCS3 regulates STAT5, which is the STAT regulator for Th17 cells, and SOCS1 regulates STAT5, which is the STAT regulator for Treg cells (Frobose et al., 2006; McBerry et al., 2012; Hovsepian et al., 2013; Yu et al., 2015). Furthermore, Yu et al. (2008) reported that overexpression of SOCS3 inhibited the inflammatory response by suppressing JAK2/STAT3 signaling in a caerulein-induced AP model both *in vitro* and *in vivo*.

MicroRNAs (miRNAs) are protein regulators that play an important role in a wide range of cellular functions. There is increasing evidence for the close relationship between miRNA expression, Th17 cell differentiation and disease pathology (Du et al., 2009; Mycko et al., 2012; Zhu et al., 2012, 2014; Murugaiyan et al., 2015). For example, miR-384 promotes Th17 cell differentiation through targeting SOCS3, leading to the pathogenesis of experimental autoimmune encephalomyelitis, an animal model of multiple sclerosis (Qu et al., 2017). The miRNA, miR-155, which was the subject of this study, has a range of known biological functions, which include the induction of Toll-like receptor (TLR) activation in monocytes/macrophages and the modulation of TLR signaling, facilitating pro-inflammatory cellular responses (Singh et al., 2014) and initiating systemic inflammatory responses (O’Connell et al., 2007; Tili et al., 2007; Pedersen and David, 2008), as well as regulating Treg cell differentiation, maintenance, and function (Cobb et al., 2006; Lu et al., 2009). The expression of this miRNA can be induced by inflammatory cytokines, such as TNFα, that are released into the circulation in the initial stages of a systemic inflammatory response (O’Connell et al., 2007; Sheedy and O’Neill, 2008) and miR-155 is considered a major inflammatory mediator that is crucial in the early stages of AP development (Sheedy and O’Neill, 2008; Kurowska-Stolarska et al., 2011; Nahid et al., 2011; Tarassishin et al., 2011; Piccinini and Midwood, 2012). In a mouse model of SAP, Tian et al. (2013) reported that miR-155 was significantly overexpressed in the intestinal epithelia, particularly during the initial inflammatory response that is characteristic of SAP (Sheedy and O’Neill, 2008; Kurowska-Stolarska et al., 2011; Nahid et al., 2011; Tarassishin et al., 2011; Piccinini and Midwood, 2012). This TNFα-regulated miR-155 overexpression inhibited the expression of the apical junction complex component protein syntheses, ZO-1 and E-cadherin, by downregulating the post-transcriptional expression of RhoA, thereby disrupting the intestinal epithelial barrier (Tian et al., 2013).

It was proposed that expression of miR-155 induced the proliferation of Treg cells via suppression of SOCS1 (Lu et al., 2009). Dudda et al. (2013) confirmed this, showing that miR-155 plays an important role in promoting CD8(+) T cell immunity, and miR-155 and its target SOCS-1 are key regulators of effector CD8(+) T cells. Furthermore, miR-155 was reported to suppress the expression of SOCS1, triggering cytokine signaling through STAT5 (Dudda et al., 2013). In another study, Yao et al. (2012) demonstrated that miR-155 enhanced Treg and Th17 cell differentiation and Th17 cell function by targeting SOCS1. We were therefore interested in studying whether the interaction between miR-155 and SOCS1 could also play a role in the disturbed regulation of the Th17/Treg ratio in SAP. We employed a well-established mouse model of AP that involves the administration of caerulein, a cholecystokinin analogue, which at low concentration induces a mild pancreatitis that resolves within a few days, or at high concentration induces a more advanced stage of the disease resembling SAP (Niederau et al., 1985). Our findings confirm the role of miR-155 as a major inflammatory mediator and offer insight into the mechanism of action of miR-155 in the disease pathology associated with SAP.

## Materials and Methods

### Patients and Ethics Statement

A total of 100 consecutive patients from the Shanghai Tenth People’s Hospital were enrolled in this study between October 2016 and October 2017. All procedures were performed according to the guidelines of the ethical committee of Tenth People’s Hospital, Tongji University, Shanghai, China, and all subjects provided informed consent to participate in the study.

All patients admitted to the hospital with a diagnosis of AP and the onset of symptoms within the last 72 h were included in this study. For each patient, the age, sex, etiological factors, body mass index (BMI), the presence/absence of organ failure and local complications, medical interventions, in-hospital mortality, and the length of hospital stay were recorded (**Table 1**).

**Table 1:** Differences in clinical course of mild, moderate, and severe AP.

Characteristic	Mild AP (*n* = 40)	Moderate AP (*n* = 40)	Severe AP (*n* = 20)
Age	52.42 ± 14.56	48 ± 18.15	51 ± 16.31
**Sex**
Male (%)	22 (36.7%)	22 (55%)	12 (60%)
Female (%)	38 (63.3%)	18 (45%)	8 (40%)
**Etiological factor**
Blood glucose (mmo/L)	7.81 ± 3.93	9.68 ± 5.31***	24.48 ± 1.89***###
AST (U/L)	120.55 ± 147.44	43.07 ± 20.89***	110.85 ± 66.13###
LDH (U/L)	810.48 ± 467.98	745.63 ± 419.38	1626.67 ± 598.78***###
WBC (10 × 10^9^/L)	11.01 ± 3.65	10.86 ± 7.46	14.91 ± 8.04*#
Ca (nmol/L)	2.12 ± 0.15	2.12 ± 0.32	1.62 ± 0.55*#
BUN (nmol/L)	5.35 ± 2.2	6.32 ± 3.97	10.7 ± 1.26*#
FT3 (pmol/L)	2.67 ± 0.48	2.49 ± 0.49	1.83 ± 1.28*#
TSH (IU/L)	0.26 ± 0.25	0.14 ± 0.15*	0.29 ± 0.07
FT4 (pmol/L)	12.45 ± 2.14	12.72 ± 4.55	7.8 ± 1.32*#
TT3 (pmol/L)	0.74 ± 0.21	0.66 ± 0.38	0.35 ± 0.22*#
TT4 (pmol/L)	78.64 ± 25.03	86.5 ± 41.82	40.5 ± 18.36***###

*AST, glutamic oxalacetic transaminase; LDH, lactate dehydrogenase; WBC, white blood cell; Ca, Serum carbohydrate antigen 199; BUN, blood urea nitrogen; FT3, free triiodothyronine; TSH, thyrotropic hormone; FT4, free tetraiodothyronine; TT3, total triiodothyronine; TT4, total tetraiodothyronine. ^∗^P < 0.05, ^∗∗^P < 0.01, ^∗∗∗^P < 0.001 vs. mild AP; ^#^P < 0.05, ^##^P < 0.01, ^###^P < 0.001 vs. severe AP*.

The diagnosis of AP was classified into three groups retrospectively as follows: mild AP (AP, 40 patients), moderate SAP (MSAP, 40 patients), and SAP (20 patients), according to the revised Atlanta 2012 classification as we previously reported (Jia et al., 2015). Peripheral blood samples from AP patients were obtained and miR-155 expression levels in serum were detected by qRT-PCR.

### Isolation and Induction of CD4^+^ T Cells

Whole-blood samples were obtained from all of the enrolled subjects. Human PBMCs were isolated from the whole blood samples by Ficoll–Hypaque density gradient centrifugation (Solarbio, Beijing, China). After isolation, the PBMCs were washed twice and resuspended in phosphate-buffered saline (PBS) to isolate CD4^+^ T lymphocytes. The CD4^+^ T cells were then isolated from the PBMCs using a magnetic cell sorting system (MACS) (Invitrogen Dynal AS, Oslo, Norway) according to the manufacturer’s instructions. The purity of CD4^+^ T lymphocytes was confirmed by fluorescence-activated cell sorting (FACS) analysis.

For Th17 differentiation, purified CD4^+^ T lymphocytes were cultured for 3 days under Th17-cell polarizing conditions: RPMI-1640 medium containing 10% fetal calf serum, 1 mM glutamine, 0.1 mM beta-mercaptoethanol, 1% non-essential amino acids

(Sigma-Aldrich, St. Louis, MO, United States), 5 ng/mL IL-2 (R&D Systems, Minneapolis, MN, United States), 20 ng/mL IL-6, 5 ng/mL transforming growth factor-b, 10 ng/mL IL-23, 2 mg/mL anti-IL-4, 2 mg/mL anti-interferon-gamma (BD Pharmingen, San Jose, CA, United States) and anti-CD3 and anti-CD28-coated beads (Invitrogen, Carlsbad, CA, United States), as previously described (Qu et al., 2017). To identify the effect of miR-155 on Th17 differentiation, miR-155 mimics were designed and synthesized by GenePharma Company (Shanghai, China) and cell transfection was performed using Lipofectamine 2000 reagent. The expression of miR-155 was detected by RT-PCR after transfection with miR-155 mimics for 48 h or miR-155 inhibitor was used to pretreat the purified CD4^+^ T lymphocytes for 24 h before the induction of Th17 polarization with/without caerulein (2 μM) induction (Chen et al., 2015).

### Detection of Th17 and Treg Cell Frequencies by Flow Cytometry

Whole blood (1 mL) was diluted 1:1 with RPMI 1640 medium. The samples were stimulated with 1 μg/mL ionomycin and 25 ng/ml phorbol myristate acetate in the presence of 1.7 mg/mL monensin (all from Sigma-Aldrich) to inhibit protein transportation and then incubated for 4 h at 37°C in an atmosphere containing 5% CO_2_. Next, the samples were stained with anti-human CD4-PE-Cy5 (BD Biosciences, San Jose, CA, United States) for 15 min. Erythrocytes were lysed with lysis buffer (BD Biosciences) and removed by washing. Leukocyte cells were then permeabilized with permeabilization solution (BD Biosciences) for 10 min. The permeabilized cells were labeled with either anti-human Foxp3-FITC or IL-17-PE antibodies (BD Biosciences), and isotype-matched control antibodies were included to rule out non-specific Fc receptor binding. Finally, the samples were washed and fixed with 1.5% paraformaldehyde prior to the analyses. Three-color flow cytometry was performed and Cell Quest Pro software (BD Biosciences) was used to determine the percentages of Th1 and Th2 cells. A gate was set on the CD4^+^ T cells, and at least 5000 cells were counted. Non-specific staining with the isotype control monoclonal antibody was less than 1%.

### Quantitative RT-PCR

Total RNA from serum, CD4^+^ T cells or pancreatic tissue was extracted using Trizol reagent (Ambion, Austin, TX, United States). Quantitative RT-PCR analyses of miR-155 levels were performed using SYBRGreen miRNA assays (Genechem, Shanghai, China) with U6 small nuclear RNA as an internal reference for normalization. The relative expression levels of miRNAs were evaluated using the 2^-ΔΔct^ method and expression levels were normalized relative to those of U6. The following primers were used: miR-155, 5^′^-CTCAACTGGTGTCGTGGAGTCGGCAATTCAGTTGAGACCCCTAT-3^′^ and 5^′^-ACACTCCAGCTGGGTTAATGCTAATCGTGAT-3^′^; and U6, 5^′^-CTCGCTTCGGCAGCACA-3^′^ and 5^′^-AACGCTTCACGAATTTGCGT-3^′^.

### Measurement of Serum Amylase, IL-6, IL-13, and TNF-α

Serum amylase was detected using a commercial kit that involved a spectrophotometric method (Nanjing Jiancheng Bioengineering Institute, Nanjing, China) according to the manufacturer’s instructions. Enzyme-linked immunosorbent assay (ELISA) kits were used to evaluate the levels of TNF-α, IL-13, and IL-6 in sera.

### Mouse Model of AP

All experiments were performed in accordance with experimental protocols approved by the Committee for Research and Animal Ethics of Tenth People’s Hospital, Tongji University, Shanghai, China. C57BL/6 mice, of 6–8 weeks of age, were purchased from the Model Animal Research Center of Nanjing University. The mice were housed at a constant temperature of 20–22°C with a 12 h:12 h light–dark cycle. Before the experiment, the animals were fed a standard rodent diet and allowed access to water. Twenty-four hours prior to the experiment, the mice were deprived of food but allowed access to water.

Eighty BALB/c mice were randomly divided into four experimental groups. Mice were injected via the intraperitoneal route with 0, 10, 50, or 250 μg/ml caerulein dissolved in sterile distilled water at a dose of 0.1 ml per day for 12 days; these groups modeled the control, AP, MAP, and SAP stages of disease severity. To investigate the effects of miR-155, approximately 2 × 10^7^ transforming units of recombinant lentiviruses were delivered by injection into the tail veins 7 days before caerulein induction (5 mice in each group). MiR-155 interference lentivirus vectors were constructed by Shanghai Genechem Company.

Mice were euthanized under anesthesia (10% chloral hydrate, 3 ml/kg body weight) by intraperitoneal injection 24 h after the injection of sodium deoxycholate, and tissue samples were collected immediately. Arterial blood samples were collected from the abdominal aorta.

### Histological Analyses

For histological analyses, pancreatic tissue from each group was fixed with 4% paraformaldehyde, then embedded in paraffin. Sections (4-μm thick) that had been deparaffinized and rehydrated were stained with hematoxylin and eosin (H&E).

### Luciferase Reporter Assay

To investigate whether miR-155 directly regulates SOCS1 expression, the sequence of the 3^′^-untranslated region (UTR) of SOCS1 was inserted downstream of a Renilla luciferase open reading frame in the pGL3-CMV vector (Promega, Madison, WI, United States). HEK293T cells were transfected with the pGL3-basic construct along with either the miR-155 mimic or a scrambled control using Lipofectamine 2000 (Invitrogen). After 24 h, the cells were harvested, and luciferase activity was measured. The results are presented as the ratio of Renilla luciferase activity to firefly luciferase activity. All of these experiments were performed at Yingbai Corporation (Shanghai, China).

### Western Blot Analysis

Western blotting was performed as previously described (Suzuki et al., 2001). After non-specific binding was blocked, the membranes were incubated with primary antibodies against SOCS1, IL-17, STAT1, pSTAT1 or GAPDH (1:1000) (Sigma–Aldrich, St. Louis, MO, United States). Immunoreactive proteins were visualized using enhanced chemiluminescence detection (Bioworld Technology, Nanjing, China). Immunoreactive labeling was analyzed using ImageJ 1.44 software and normalized to GAPDH protein levels.

### Statistical Analysis

Statistical analysis was carried out using the SPSS software package (version 17; IBM Corporation, Armonk, NY, United States). All of the descriptive variables were expressed as the mean ± SD. Comparisons between two groups were performed using the Student’s *t*-test, and comparisons between more than two groups employed Tukey’s multiple comparisons test. *P* < 0.05 was considered significant.

## Results

### MiR-155 Regulates AP Pathogenesis

**FIGURE 1:**
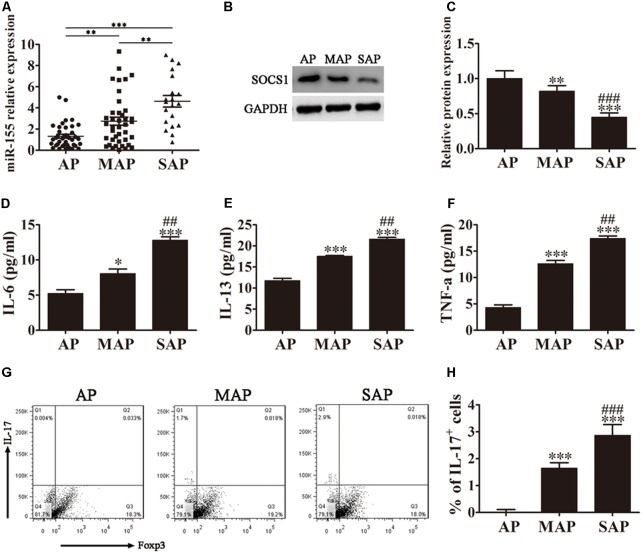
MiR-155 regulates AP pathogenesis. **(A)** Quantitative RT-PCR analyses of miR-155 expression in the sera of AP patients. Data are presented as the mean ± SD. ^∗∗^*P* < 0.01, ^∗∗∗^*P* < 0.001. **(B)** Western blot analysis of SOCS1 expression in CD4^+^ T cells isolated from the different groups of AP patients. GAPDH was detected as an endogenous control. **(C)** The relative SOCS1 expression was determined by densitometric analysis. Data are presented as the mean ± SD. ^∗∗^*P* < 0.01, ^∗∗∗^*P* < 0.001 vs. AP group. ^###^*P* < 0.001 vs. MAP group. **(D–F)** ELISA to determine the concentrations of inflammatory factors IL-6, IL-13 and TNF-α in the sera from the different groups of AP patients. Data are presented as the mean ± SD. ^∗^*P* < 0.05, ^∗∗∗^*P* < 0.001 vs. AP group. ^##^*P* < 0.01 vs. MAP group. **(G)** Representative flow cytometric analyses of IL-17 and Foxp3 cells among CD4^+^ gated cells from different groups of AP patients. **(H)** The percentage of IL-17^+^ cells. Data are presented as the mean ± SD. ^∗∗∗^*P* < 0.001 vs. AP group. ^###^*P* < 0.01 vs. MAP group. AP, mild AP; MAP, moderate AP; SAP, severe AP.

The diagnoses of the patients enrolled in this study were classified into three groups retrospectively: mild (AP), moderate (MAP), and severe (SAP), according to the revised Atlanta 2012 classification. To determine whether miR-155 expression levels differed with disease severity, quantitative RT-PCR analyses of miR-155 expression in the serum of patients diagnosed with AP were performed. As shown in **Figure 1A**, miR-155 expression levels positively correlated with disease severity, with the SAP group of patients displaying the highest miR-155 expression.

SOCS1 has previously been reported to be a target for suppression by miR-155 (Lu et al., 2009). We therefore analyzed SOCS1 expression in the CD4^+^ T lymphocytes isolated and purified from the whole blood of AP patients by western blot analysis (**Figure 1B**). The relative SOCS1 expression decreased with increased disease severity, with the SAP group of patients displaying the lowest levels of SOCS1 expression (*P* < 0.001 vs. AP group, *P* < 0.001 vs. MAP group).

SOCS1 suppresses cytokine signaling via modulation of the STAT pathway (Fujimoto and Naka, 2010; Wu et al., 2014). We therefore performed ELISA on the sera from the three groups of AP patients to determine the concentrations of inflammatory factors IL-6, IL-13 and TNF-α (**Figures 1D–F**). The results showed a clear increase in all three inflammatory factors analyzed corresponding to disease severity, with the sera from patients diagnosed with SAP exhibiting the highest levels of IL-6, IL-13, and TNF-α (*P* < 0.001 vs. AP group, *P* < 0.01 vs. MAP group).

The accumulation of IL-17-producing CD4^+^ T helper (Th17) cells has been linked to AP pathogenesis, and is thought to significantly contribute to the inflammatory response. We next investigated the Th17/Treg ratio in the AP patients by flow cytometric analyses of CD4^+^ gated cells isolated from the whole blood of the different groups of AP patients using antibodies against IL-17 and Foxp3 (**Figure 1G**). Foxp3 is a specific marker of CD4^+^ regulatory T cells, and an IL-17 antibody was used to detect Th17 cells. Our results demonstrated that the percentage of IL-17^+^ cells was significantly increased with increasing disease severity, indicating an elevated Th17/Treg ratio (**Figure 1H**). The patients diagnosed with SAP exhibited the highest percentage of IL-17^+^ cells (*P* < 0.001 vs. AP group, *P* < 0.01 vs. MAP group).

Taken together, these findings indicate that miR-155 expression levels positively correlate with AP disease severity and expression of the miR-155 target, SOCS1, negatively correlates with AP pathogenesis. The severity of AP, and the corresponding downregulation of SOCS1, related to an increase in the concentration of inflammatory factors IL-6, IL-13 and TNF-α, and the percentage of IL-17^+^ cells.

### MiR-155 Promotes the Generation of Th17 Cells

**FIGURE 2:**
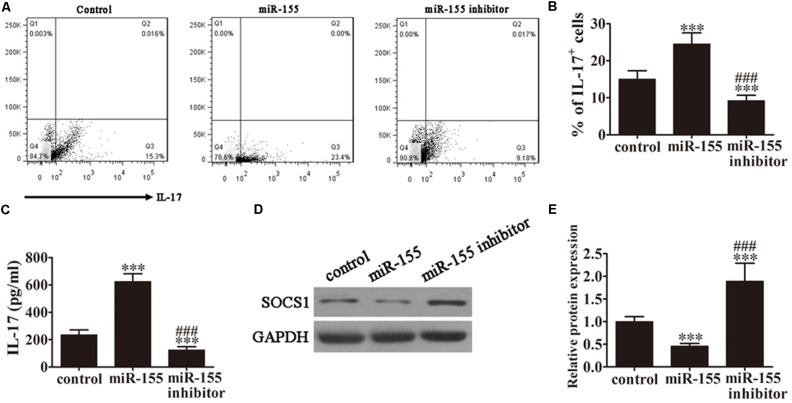
MiR-155 promotes the generation of Th17 cells. CD4^+^ T cells from AP patients were first pretreated with miR-155 mimics or miR-155 inhibitor for 24 h. **(A)** Flow cytometric analyses of CD4^+^ T cells after 3 days of induction under Th17-polarizing conditions. **(B)** The percentage of IL-17^+^ cells. Data are presented as the mean ± SD. ^∗∗∗^*P* < 0.001 vs. control. ^###^*P* < 0.01 vs. miR-155 overexpression group. **(C)** ELISA to detect IL-17 in the culture supernatants of the pretreated CD4^+^ T cells (*n* = 5 per group). Data are presented as the mean ± SD. ^∗∗∗^*P* < 0.001 vs. control. ^###^*P* < 0.01 vs. miR-155 overexpression group. **(D)** Western blot analysis of SOCS1 expression in the pretreated CD4^+^ T cells (*n* = 3). GAPDH was detected as an endogenous control. **(E)** The relative SOCS1 expression was determined by densitometric analysis. Data are presented as the mean ± SD. ^∗∗∗^*P* < 0.001 vs. control. ^###^*P* < 0.01 vs. miR-155 overexpression group.

To analyze the role of miR-155 in the accumulation of IL-17^+^ cells, CD4^+^ T cells isolated from AP patients were pretreated with miR-155 mimics or miR-155 inhibitor for 24 h prior to analysis. Then, following 3 days of induction under Th17-polarizing conditions, flow cytometric analyses were performed on these CD4^+^ T cells (**Figure 2A**). The percentage of IL-17^+^ cells was significantly increased in those cells in which miR-155 was overexpressed and decreased in cells in which miR-155 was inhibited compared with the control (*P* < 0.001; **Figure 2B**), indicating that miR-155 promotes the generation of Th17 cells.

To determine the levels of IL-17 production in response to miR-155 overexpression, ELISA of IL-17 was performed on the culture supernatants of the pretreated CD4^+^ T cells from AP patients (**Figure 2C**). MiR-155 overexpression upregulated IL-17 levels in the cell supernatants (*P* < 0.001 vs. control), and pretreatment with the miR-155 inhibitor reversed this trend (*P* < 0.001 vs. control, *P* < 0.01 vs. miR-155 overexpression group).

Next, we investigated SOCS1 expression by western blot analysis of CD4^+^ T cells isolated from the AP patients (**Figure 2D**). SOCS1 protein expression was significantly decreased in miR-155 overexpressing cells (*P* < 0.001 vs. control), and this trend was not only reversed following pretreatment with the miR-155 inhibitor but SOCS1 protein expression was significantly increased compared with the control (*P* < 0.001 vs. control, *P* < 0.01 vs. miR-155 overexpression group; **Figure 2E**). This not only provided further evidence of the suppression of SOCS1 by miR-155 and the resulting upregulation of the inflammatory response, but also indicated that inhibition of miR-155 not only restored SOCS1 levels but positively upregulated SOCS1 expression.

### Inhibition of miR-155 Expression Decreased the Conversion of CD4^+^ T Cells to Th17 Cells After Caerulein Induction

**FIGURE 3:**
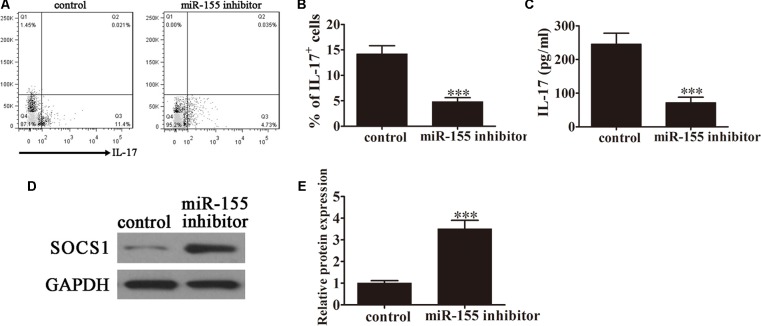
Inhibition of miR-155 expression decreased the conversion of CD4^+^ T cells to Th17 cells following caerulein induction. CD4^+^ T cells were first induced with caerulein (2 μM) to simulate AP for 48 h and were pretreated with or without miR-155 inhibitor. **(A)** Flow cytometric analyses of IL-17 cells (*n* = 3). **(B)** The percentage of IL-17^+^ cells. Data are presented as the mean ± SD. ^∗∗∗^*P* < 0.001 vs. control. **(C)** ELISA of IL-17 in the culture supernatants (*n* = 5 per group). Data are presented as the mean ± SD. ^∗∗∗^*P* < 0.001 vs. control. **(D)** Western blot analysis of the expression of SOCS1 in the pretreated CD4^+^ T cells (*n* = 3). GAPDH was detected as an endogenous control. **(E)** The relative SOCS1 expression was determined by densitometric analysis. Data are presented as the mean ± SD. ^∗∗∗^*P* < 0.001 vs. control.

Caerulein induction is a well-established method of stimulating the pathology of AP. To investigate the role of miR-155 in modulating Th17 cells, CD4^+^ T cells were pretreated with caerulein (2 μM) for 48 h following treatment with the miR-155 inhibitor, then flow cytometric analyses were performed (**Figure 3A**). The percentage of IL-17^+^ cells was significantly decreased in the AP-induced cells treated with the inhibitor (*P* < 0.001 vs. control; **Figure 3B**), further confirming the role of miR-155 in modulating the Th17 ratio. ELISA to detect IL-17 expression levels in the culture supernatants revealed downregulation of IL-17 in the absence of miR-155 in AP-induced cells (*P* < 0.001 vs. control; **Figure 3C**). Furthermore, western blot analysis demonstrated significantly increased SOCS1 expression in AP-induced cells following treatment with the inhibitor (*P* < 0.001 vs. control; **Figures 3D,E**). Taken together, these findings confirmed that inhibition of miR-155 expression decreased the conversion of CD4^+^ T cells to Th17 cells after caerulein induction, thereby decreasing IL-17 production, with a simultaneous increase in SOCS1 expression.

### SOCS1 Is a Direct Target of miR-155

**FIGURE 4:**
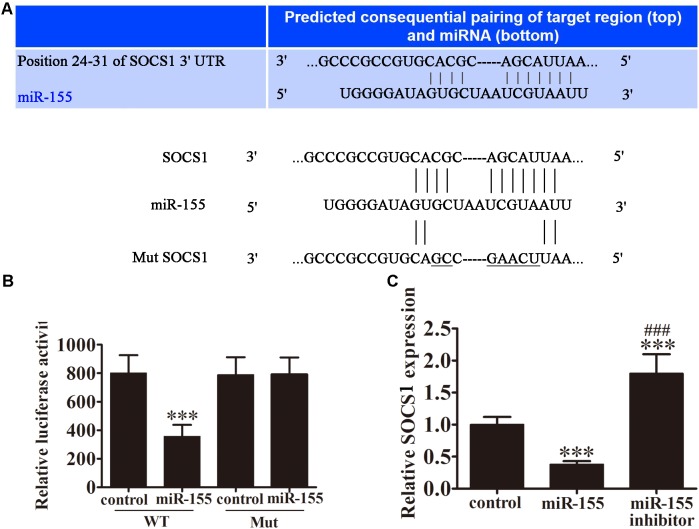
SOCS1 is a direct target of miR-155. **(A)** Illustration of the sequence match between miR-155 and SOCS1 mRNA determined using TargetScan. **(B)** Luciferase activity of reporter vectors containing wild-type (WT) or mutated (MUT) SOCS1 3^′^-UTR co-transfected with miR-155 or control miR (ctrl; *n* = 3 per group). Data are presented as means ± SD. ^∗∗∗^*P* < 0.001 vs. control group. **(C)** Rt-PCR analyses for SOCS1 in CD4^+^ T cells 3 days post-transfection (*n* = 3 per group). Data are presented as means ± SD. ^∗∗∗^*P* < 0.001 vs. control group. ^###^*P* < 0.001 vs. miR-155 group.

To investigate the potential target genes of miR-155, bioinformatic analysis was performed using the TargetScan software. Using this approach, an miR-155 binding site was predicted in the 3^′^-UTR of SOCS1 mRNA (**Figure 4A**). To examine the regulation of SOCS1 by miR-155, we performed a dual-luciferase reporter assay in CD4^+^ T cells. Cells were co-transfected with wild-type (WT) or mutant (MUT) SOCS1 3^′^-UTR plasmids, and miR-155 or control miR. MiR-155 significantly inhibited the luciferase reporter activity of the WT (*P* < 0.001 vs. control group) but not the MUT SOCS1 3^′^-UTR, indicating that SOCS1 is a direct target of miR-155 (**Figure 4B**). RT-PCR analysis of SOCS1 in miR-155-transfected cells confirmed that this miRNA downregulated the expression of SOCS1 in CD4^+^ T cells (*P* < 0.001 vs. control, **Figure 4C**). This effect was reversed by treatment of the cells with miR-155 inhibitor (*P* < 0.001 vs. control, *P* < 0.001 vs. miR-155 group; **Figure 4C**).

### The Effect of miR-155 on Histological Changes in the Pancreas

**FIGURE 5:**
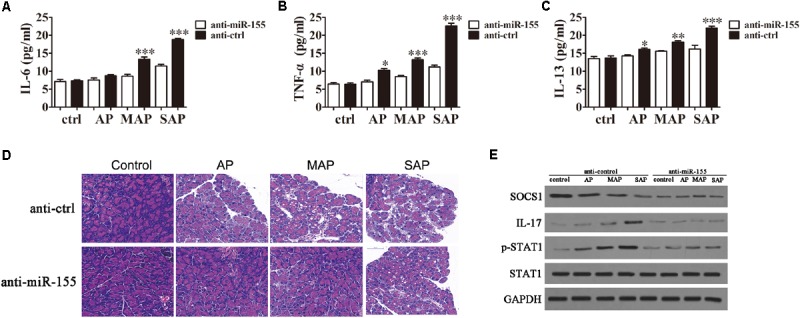
The effect of miR-155 on histological changes in the pancreas. BALB/c mice were injected with miR-155 interference lentiviruses (anti-miR-155) or control lentiviruses (anti-ctrl) followed by 10, 50 or 250 μg/ml caerulein to model the AP, MAP and SAP stages of disease severity. **(A–C)** The expression of inflammatory factors IL-6 **(A)**, TNF-α **(B)** and IL-13 **(C)** was measured by ELISA (*n* = 5). Data are presented as the mean ± SD. ^∗^*P* < 0.05, ^∗∗^*P* < 0.01, ^∗∗∗^*P* < 0.001 vs. anti-ctrl group. **(D)** HE staining of the pancreatic tissue. **(E)** Western blot analysis of the expression levels of SOCS1, IL-17, STAT1, and pSTAT1 (*n* = 3). GAPDH was detected as an endogenous control.

To examine the effects of miR-155 on inflammatory cytokine expression in the pancreatic tissue of mice, the animals were injected with miR-155 interference lentiviruses followed by 10, 50, or 250 μg/ml caerulein to model the AP, MAP and SAP stages of disease severity. ELISA revealed that in the miR-155-silenced pancreatic tissue the production of inflammatory cytokines, IL-6 (**Figure 5A**), TNF-α (**Figure 5B**) and IL-13 (**Figure 5C**), was decreased compared with those injected with the control lentiviruses. This effect became more pronounced as the disease stage progressed. Similarly, HE staining of the pancreatic tissue from the different disease stages in the mice injected with control lentiviruses revealed slight interstitial edema and low levels of inflammatory cell infiltration in the AP mice progressing to broad necrosis of acinar cells and interstitial edema in SAP mice (**Figure 5D**). Whereas in the mice injected with miR-155 interference lentiviruses, the pathogenic condition was attenuated at all disease stages revealing an improvement in the pathology of the pancreas compared with the control. Western blot analysis confirmed downregulation of SOCS1, IL-17, and pSTAT1 in response to miR-155 silencing compared with control mice throughout the course of the disease (**Figure 5E**). These findings clearly indicated that miR-155 plays a crucial role in the progression of AP disease via a mechanism that involves the induction of an inflammatory response, regulation of the Th17/Treg ratio, and targeting of SOCS1 via activation of the STAT signaling pathway.

## Discussion

SAP is a serious, often lethal, condition associated with intestinal barrier dysfunction resulting from apical junction complex damage. This cellular injury is caused by the dysregulation of digestive enzymes leading to autodigestion of the pancreas, which in turn leads to the accumulation of Th17 cells and the induction of a significant inflammatory response, which is a hallmark of SAP. The interaction between miR-155 and SOCS1 has previously been reported, as has the resulting perturbation of differentiated T cell populations. However, this remained to be experimentally demonstrated in SAP. Here, we performed *in vitro* assays using CD4^+^ T cells isolated from AP patients and *in vivo* assays using a mouse model of caerulein-induced AP, to analyze the mechanism by which the upregulation of miR-155 contributes to SAP. Our findings confirmed that miR-155 mediated upregulation of the Th17/Treg ratio and inflammatory cytokine expression levels via the suppression of SOCS1 in experimental AP.

In this study, SOCS1 was identified as one of the target genes of miR-155, confirming the findings of a previous report (Lu et al., 2009). Further to this, we provided evidence that miR-155 binds directly to the 3^′^-UTR of SOCS1. Our findings clearly indicated that miR-155 expression and the inflammatory response correlate positively with the severity of AP, while they correlate negatively with the levels of SOCS1. This was in line with previous findings for miR-155 in other inflammatory disease or animal models (Dudda et al., 2013; Singh et al., 2014). For example, Dudda et al. (2013) reported that miR-155 and its target SOCS1 were key regulators of effector CD8(+) T cells, thereby affecting cytokine signaling through STAT5. Similarly, Singh et al. (2014) previously demonstrated the role for miR-155 in facilitating pro-inflammatory cellular responses during experimental colitis in mice by reducing Th1/Th17 responses.

In the current study, in addition to showing that miR-155 overexpression dysregulated *SOCS1* gene expression at the post-transcriptional level, interestingly, we also showed that miR-155 inhibition not only restored SOCS1 protein expression but actually upregulated its expression. This suggested that miR-155 may compete with another regulatory factor that positively affects SOCS1 expression, although further studies will be required to confirm this. Investigations will also be needed to analyze the potential clinical implications of upregulated SOCS1 expression in the absence of miR-155 if this microRNA is to be targeted therapeutically.

The SOCS family of proteins are cytokine-inducible negative regulators of cytokine signaling that operate by a negative feedback mechanism. The SOCS proteins are reported to modulate cytokine signaling via the JAK/STAT pathway (Fujimoto and Naka, 2010; Wu et al., 2014). The interactions between SOCS proteins and STAT regulators is complex, with different SOCS proteins modulating cytokine signaling via different STAT regulators. The role of SOCS3 in mediating the inflammatory response during SAP has previously been reported (Yu et al., 2008). In our study, the suppression of SOCS1 by miR-155 during the initial stages of SAP was found to lead to upregulation of the Th17/Treg ratio and inflammatory cytokine expression levels via a mechanism involving the JAK/STAT pathway, in line with previous findings (Singh et al., 2014; Qu et al., 2017). These results were expected since Th17 cell accumulation and upregulation of pro-inflammatory cytokines in the pancreas are well recognized features of AP pathogenesis. However, only Th17 cell numbers were assessed in this study, future studies will be needed to measure Treg cell numbers and confirm any alterations since it has been suggested in the literature that SOCS1 inhibition upregulates Treg cells.

Limited treatment modalities are currently available for SAP. Identification of a therapeutic strategy to limit local inflammation and prevent its progression to severe disease is therefore crucial to limit the mortality associated with pancreatitis. The role of miRNAs in disease pathogenesis is a rapidly expanding field of research and a promising potential source of new therapeutic targets. Our findings provide strong evidence that miR-155 is increased in AP patients, and its concentration increases with disease severity. Furthermore, inhibition of miR-155 in a mouse model of AP led to better disease outcomes. To our knowledge, this is the first study to demonstrate that the interaction between miR-155 and SOCS1 plays a role in the disturbed regulation of the Th17/Treg ratio in SAP and demonstrates that this microRNA is therefore a promising candidate biomarker and/or therapeutic target for the treatment of SAP.

## Author Contributions

DW, YL, and YZ designed the studies and prepared the manuscript with comments from all authors. MT, PZ, and HL performed all the experiments and analyzed the data. TZ and YL carried out all the experiments and revised the manuscript.

## Conflict of Interest Statement

The authors declare that the research was conducted in the absence of any commercial or financial relationships that could be construed as a potential conflict of interest.

## References

[B1] Ammori B. J. (2003). Role of the gut in the course of severe acute pancreatitis. *Pancreas* 26 122–129. 10.1097/00006676-200303000-00006 12604908

[B2] Ammori B. J. Leeder P. C. King R. F. Barclay G. R. Martin I. G. Larvin M. (1999). Early increase in intestinal permeability in patients with severe acute pancreatitis: correlation with endotoxemia, organ failure, and mortality. *J. Gastrointest. Surg.* 3 252–262. 10.1016/S1091-255X(99)80067-5 10481118

[B3] Bhatia M. (2004). Apoptosis versus necrosis in acute pancreatitis. *Am. J. Physiol. Gastrointest. Liver Physiol.* 286 G189–G196. 10.1152/ajpgi.00304.2003 14715516

[B4] Capurso G. Zerboni G. Signoretti M. Valente R. Stigliano S. Piciucchi M. (2012). Role of the gut barrier in acute pancreatitis. *J. Clin. Gastroenterol.* 46(Suppl.), S46–S51. 10.1097/MCG.0b013e3182652096 22955357

[B5] Chen Y. Li L. Lu Y. Su Q. Sun Y. Liu Y. (2015). Upregulation of miR-155 in CD4(+) T cells promoted Th1 bias in patients with unstable angina. *J. Cell. Physiol.* 230 2498–2509. 10.1002/jcp.24987 25760478

[B6] Clark J. A. Coopersmith C. M. (2007). Intestinal crosstalk: a new paradigm for understanding the gut as the “motor” of critical illness. *Shock* 28 384–393. 10.1097/shk.0b013e31805569df 17577136 PMC2084394

[B7] Cobb B. S. Hertweck A. Smith J. O’Connor E. Graf D. Cook T. (2006). A role for Dicer in immune regulation. *J. Exp. Med.* 203 2519–2527. 10.1084/jem.20061692 17060477 PMC2118134

[B8] Du C. Liu C. Kang J. Zhao G. Ye Z. Huang S. (2009). MicroRNA miR-326 regulates TH-17 differentiation and is associated with the pathogenesis of multiple sclerosis. *Nat. Immunol.* 10 1252–1259. 10.1038/ni.1798 19838199

[B9] Dudda J. C. Salaun B. Ji Y. Palmer D. C. Monnot G. C. Merck E. (2013). MicroRNA-155 is required for effector CD8+ T cell responses to virus infection and cancer. *Immunity* 38 742–753. 10.1016/j.immuni.2012.12.006 23601686 PMC3788592

[B10] Frobose H. Rønn S. G. Heding P. E. Mendoza H. Cohen P. Mandrup-Poulsen T. (2006). Suppressor of cytokine signaling-3 inhibits interleukin-1 signaling by targeting the TRAF-6/ TAK1 complex. *Mol. Endocrinol.* 20 1587–1596. 10.1210/me.2005-0301 16543409

[B11] Fujimoto M. Naka T. (2010). SOCS1, a negative regulator of cytokine signals and TLR responses, in human liver diseases. *Gastroenterol. Res. Pract.* 2010:470468. 10.1155/2010/470468 20862390 PMC2939392

[B12] Hovsepian E. Penas F. Siffo S. Mirkin G. A. Goren N. B. (2013). IL-10 inhibits the NF-kappaB and ERK/MAPK-mediated production of pro-inflammatory mediators by up-regulation of SOCS-3 in *Trypanosoma cruzi*-infected cardiomyocytes. *PLoS One* 8:e79445. 10.1371/journal.pone.0079445 24260222 PMC3832617

[B13] Jamshidian A. Kazemi M. Shaygannejad V. Salehi M. (2015). The effect of plasma exchange on the expression of FOXP3 and RORC2 in relapsed multiple sclerosis patients. *Iran. J. Immunol.* 12 311–318. 26714422 10.22034/iji.2015.16759

[B14] Jamshidian A. Shaygannejad V. Pourazar A. Zarkesh-Esfahani S. H. Gharagozloo M. (2013). Biased Treg/Th17 balance away from regulatory toward inflammatory phenotype in relapsed multiple sclerosis and its correlation with severity of symptoms. *J. Neuroimmunol.* 262 106–112. 10.1016/j.jneuroim.2013.06.007 23845464

[B15] Jia R. Tang M. Qiu L. Sun R. Cheng L. Ma X. (2015). Increased interleukin-23/17 axis and C-reactive protein are associated with severity of acute pancreatitis in patients. *Pancreas* 44 321–325. 10.1097/MPA.0000000000000284 25426616

[B16] Juvonen P. O. Alhava E. M. Takala J. A. (2000). Gut permeability in patients with acute pancreatitis. *Scand. J. Gastroenterol.* 35 1314–1318. 10.1080/003655200453683 11199373

[B17] Kurowska-Stolarska M. Alivernini S. Ballantine L. E. Asquith D. L. Millar N. L. Gilchrist D. S. (2011). MicroRNA-155 as a proinflammatory regulator in clinical and experimental arthritis. *Proc. Natl. Acad. Sci. U.S.A.* 108 11193–11198. 10.1073/pnas.1019536108 21690378 PMC3131377

[B18] Lochner M. Wang Z. Sparwasser T. (2015). The special relationship in the development and function of T helper 17 and regulatory T cells. *Prog. Mol. Biol. Transl. Sci.* 136 99–129. 10.1016/bs.pmbts.2015.07.013 26615094

[B19] Lu L. F. Thai T. H. Calado D. P. Chaudhry A. Kubo M. Tanaka K. (2009). Foxp3-dependent microRNA155 confers competitive fitness to regulatory T cells by targeting SOCS1 protein. *Immunity* 30 80–91. 10.1016/j.immuni.2008.11.010 19144316 PMC2654249

[B20] McBerry C. Gonzalez R. M. Shryock N. Dias A. Aliberti J. (2012). SOCS2-induced proteasomedependent TRAF6 degradation: a common anti-inflammatory pathway for control of innate immune responses. *PLoS One* 7:e38384. 10.1371/journal.pone.0038384 22693634 PMC3367914

[B21] Murugaiyan G. da Cunha A. P. Ajay A. K. Joller N. Garo L. P. Kumaradevan S. (2015). MicroRNA-21 promotes Th17 differentiation and mediates experimental autoimmune encephalomyelitis. *J. Clin. Invest.* 125 1069–1080. 10.1172/JCI74347 25642768 PMC4362225

[B22] Mycko M. P. Cichalewska M. Machlanska A. Cwiklinska H. Mariasiewicz M. Selmaj K. W. (2012). MicroRNA-301a regulation of a T-helper17 immune response controls autoimmune demyelination. *Proc. Natl. Acad. Sci. U.S.A.* 109 E1248–E1257. 10.1073/pnas.1114325109 22517757 PMC3356660

[B23] Nahid M. A. Satoh M. Chan E. K. (2011). MicroRNA in TLR signaling and endotoxin tolerance. *Cell. Mol. Immunol.* 8 388–403. 10.1038/cmi.2011.26 21822296 PMC3618661

[B24] Niederau C. Ferrell L. D. Grendell J. H. (1985). Caerulein-induced acute necrotizing pancreatitis in mice: protective effects of proglumide, benzotript, and secretin. *Gastroenterology* 88 1192–1204. 10.1016/S0016-5085(85)80079-2 2984080

[B25] Norman M. (1998). The role of cytokines in the pathogenesis of acute pancreatitis. *Am. J. Surg.* 175 76–83. 10.1016/S0002-9610(97)00240-7 9445247

[B26] O’Connell R. M. Taganov K. D. Boldin M. P. Cheng G. Baltimore D. (2007). MicroRNA-155 is induced during the macrophage inflammatory response. *Proc. Natl. Acad. Sci. U.S.A.* 104 1604–1609. 10.1073/pnas.0610731104 17242365 PMC1780072

[B27] Pedersen I. David M. (2008). MicroRNAs in the immune response. *Cytokine* 43 391–394. 10.1016/j.cyto.2008.07.016 18701320 PMC3642994

[B28] Piccinini A. M. Midwood K. S. (2012). Endogenous control of immunity against infection: tenascin-C regulates TLR4-mediated inflammation via microRNA-155. *Cell. Rep.* 2 914–926. 10.1016/j.celrep.2012.09.005 23084751 PMC3607221

[B29] Qu X. Han J. Zhang Y. Wang Y. Zhou J. Fan H. (2017). MiR-384 regulates the Th17/Treg ratio during experimental autoimmune encephalomyelitis pathogenesis. *Front. Cell. Neurosci.* 11:88. 10.3389/fncel.2017.00088 28400721 PMC5368215

[B30] Rahman S. H. Ammori B. J. Holmfield J. Larvin M. McMahon M. J. (2003). Intestinal hypoperfusion contributes to gut barrier failure in severe acute pancreatitis. *J. Gastrointest. Surg.* 7 26–35. 10.1016/S1091-255X(02)00090-2 12559182

[B31] Sheedy F. J. O’Neill L. A. (2008). Adding fuel to fire: microRNAs as a new class of mediators of inflammation. *Ann. Rheum. Dis.* 67(Suppl. 3), iii50–iii55. 10.1136/ard.2008.100289 19022814

[B32] Singh U. P. Murphy A. E. Enos R. T. Shamran H. A. Singh N. P. Guan H. (2014). miR-155 deficiency protects mice from experimental colitis by reducing T helper type 1/type 17 responses. *Immunology* 143 478–489. 10.1111/imm.12328 24891206 PMC4212960

[B33] Suzuki A. Hanada T. Mitsuyama K. Yoshida T. Kamizono S. Hoshino T. (2001). CIS3/SOCS3/SSI3 plays a negative regulatory role in STAT3 activation and intestinal inflammation. *J. Exp. Med.* 193 471–481. 10.1084/jem.193.4.471 11181699 PMC2195913

[B34] Tarassishin L. Loudig O. Bauman A. Shafit-Zagardo B. Suh H. S. Lee S. C. (2011). Interferon regulatory factor 3 inhibits astrocyte inflammatory gene expression through suppression of the proinflammatory miR-155 and miR-155^∗^. *Glia* 59 1911–1922. 10.1002/glia.21233 22170100 PMC3241213

[B35] Tian R. Wang R. L. Xie H. Jin W. Yu K. L. (2013). Overexpressed miRNA-155 dysregulates intestinal epithelial apical junctional complex in severe acute pancreatitis. *World J. Gastroenterol.* 19 8282–8291. 10.3748/wjg.v19.i45.8282 24363519 PMC3857451

[B36] Tili E. Michaille J. J. Cimino A. Costinean S. Dumitru C. D. Adair B. (2007). Modulation of miR-155 and miR-125b levels following lipopolysaccharide/TNF-alpha stimulation and their possible roles in regulating the response to endotoxin shock. *J. Immunol.* 179 5082–5089. 10.4049/jimmunol.179.8.5082 17911593

[B37] Tzartos J. S. Friese M. A. Craner M. J. Palace J. Newcombe J. Esiri M. M. (2008). Interleukin-17 production in central nervous system-infiltrating T cells and glial cells is associated with active disease in multiple sclerosis. *Am. J. Pathol.* 172 146–155. 10.2353/ajpath.2008.070690 18156204 PMC2189615

[B38] Venkatesha S. H. Dudics S. Weingartner E. So E. C. Pedra J. Moudgil K. D. (2015). Altered Th17/Treg balance and dysregulated IL-1β response influence susceptibility/resistance to experimental autoimmune arthritis. *Int. J. Immunopathol. Pharmacol.* 28 318–328. 10.1177/0394632015595757 26227656 PMC8039840

[B39] Wu B. U. Banks P. A. (2013). Clinical management of patients with acute pancreatitis. *Gastroenterology* 144 1272–1281. 10.1053/j.gastro.2013.01.075 23622137

[B40] Wu J. Ma C. Wang H. Wu S. Xue G. Shi X. (2014). A MyD88-JAK1-STAT1 complex directly induces SOCS-1expression in macrophages infected with Group A Streptococcus. *Cell. Mol. Immunol.* 12 373–383. 10.1038/cmi.2014.107 25399770 PMC4654310

[B41] Yao R. Ma Y. L. Liang W. Li H. H. Ma Z. J. Yu X. (2012). MicroRNA-155 modulates Treg and Th17 cells differentiation and Th17 cell function by targeting SOCS1. *PLoS One* 7:e46082. 10.1371/journal.pone.0046082 23091595 PMC3473054

[B42] Yu H. Liu Y. McFarland B. C. Deshane J. S. Hurst D. R. Ponnazhagan S. (2015). SOCS3 deficiency in myeloid cells promotes tumor development: involvement of STAT3 activation and myeloid-derived suppressor cells. *Cancer Immunol. Res.* 3 727–740. 10.1158/2326-6066.CIR-15-0004 25649351 PMC4570503

[B43] Yu J. H. Kim K. H. Kim H. (2008). SOCS3 and PPAR-gamma ligands inhibit the expression of IL-6 and TGF-beta1 by regulating JAK2/STAT3 signaling in pancreas. *Int. J. Biochem. Cell. Biol.* 40 677–688. 10.1016/j.biocel.2007.10.007 18035585

[B44] Zhu E. Wang X. Zheng B. Wang Q. Hao J. Chen S. (2014). miR-20b suppresses Th17 differentiation and the pathogenesis of experimental autoimmune encephalomyelitis by targeting RORγt and STAT3. *J. Immunol.* 192 5599–5609. 10.4049/jimmunol.1303488 24842756

[B45] Zhu S. Pan W. Song X. Liu Y. Shao X. Tang Y. (2012). The microRNA miR-23b suppresses IL-17-associated autoimmune inflammation by targeting TAB2, TAB3 and IKK-α. *Nat. Med.* 18 1077–1086. 10.1038/nm.2815 22660635

